# Prevalence of asymptomatic meibomian gland dysfunction in the general adult population: a systematic review and meta-analysis

**DOI:** 10.3389/fmed.2026.1797225

**Published:** 2026-04-20

**Authors:** Zhanar Abu, Kairolla Rakhimov, Maiya Taushanova, Indira Karibayeva

**Affiliations:** 1 Department of Clinical Pharmacology, Asfendiyarov Kazakh National Medical University, Almaty, Kazakhstan; 2 Center for Nursing Excellence, West Kazakhstan Marat Ospanov Medical University, Aktobe, Kazakhstan; 3 Department of Research Management, JSC Research Institute of Cardiology and Internal Diseases, Almaty, Kazakhstan; 4 Department of Health Policy and Community Health, Jiann-Ping Hsu College of Public Health, Georgia Southern University, Statesboro, GA, United States

**Keywords:** asymptomatic meibomian gland dysfunction, dry eye disease, meta-analysis, prevalence, systematic review

## Abstract

**Background/objectives:**

Meibomian gland dysfunction (MGD) constitutes a highly prevalent ocular surface condition and is a major etiological factor in tear film instability and evaporative dry eye disease. Although MGD can be objectively identified based on structural and functional abnormalities of the meibomian glands, epidemiological studies rarely differentiate between symptomatic and asymptomatic disease. Asymptomatic meibomian gland dysfunction, characterized by structural or functional gland alterations in the absence of self-reported ocular symptoms, may constitute an early and frequently overlooked phase within the disease spectrum. The objective of this systematic review and meta-analysis was to quantify the prevalence of asymptomatic MGD among adult populations.

**Methods:**

A comprehensive literature search was performed across PubMed, Web of Science, Scopus, ScienceDirect, and Google Scholar in accordance with the PRISMA guidelines. The review protocol was registered in PROSPERO (CRD420261283795). Cross-sectional and observational studies reporting the prevalence of asymptomatic MGD in adult populations were included. Data extraction and study selection were performed independently by two reviewers. A random-effects meta-analysis of proportions with logit transformation was applied using R software. Interstudy heterogeneity was quantified using the I^2^ statistic, and possible contributors to variability were investigated through sensitivity analyses and meta-regression. Methodological quality and risk of bias were assessed using a modified version of the Newcastle-Ottawa Scale, while the overall certainty of the evidence was evaluated according to the GRADE approach.

**Results:**

Eight cross-sectional studies published between 2012 and 2023 were included, comprising a total of 3,637 participants and 1,313 cases of asymptomatic MGD. The combined prevalence of asymptomatic MGD was 72.86% (95% CI: 19.33–96.78%), with substantial heterogeneity across studies (*I*^2^ = 98.8%). Sensitivity analyses identified one influential study; however, the overall finding of a high prevalence remained consistent. Meta-regression showed that sex distribution significantly contributed to between-study heterogeneity, while no association with year of publication was observed. The level of certainty for the pooled prevalence estimate was classified as low.

**Conclusion:**

Asymptomatic MGD is highly prevalent among adult populations worldwide. These findings indicate that reliance on symptom-based assessment alone may underestimate the burden of early MGD. Early identification of asymptomatic gland abnormalities may support preventive approaches to reduce progression to symptomatic dry eye disease, although further standardized and prospective studies are required.

**Systematic review registration:**

https://www.crd.york.ac.uk/PROSPERO/view/CRD420261283795, CRD420261283795.

## 1 Introduction

Meibomian gland dysfunction (MGD) represents a highly prevalent ocular surface condition involving chronic meibomian gland abnormalities that disrupt tear film homeostasis and promote ocular discomfort, inflammation, and ocular surface disease, including dry eye syndrome. Recent TFOS DEWS III reports further emphasize the multifactorial nature of MGD and provide updated insights into its pathophysiology, including gland obstruction, altered lipid composition, inflammatory processes, and tear film instability (1–4). Large-scale epidemiological investigations and systematic reviews have consistently demonstrated that MGD affects a substantial proportion of the adult population across diverse geographic regions and ethnic groups, underscoring its importance as a global public health issue (5). Although MGD is highly prevalent, it represents a heterogeneous disorder, with wide variation in reported prevalence estimates. This variability is largely driven by differences in diagnostic definitions, study designs, population characteristics, and methods of clinical assessment (6).

Current epidemiological evidence indicates that the pooled global prevalence of MGD exceeds one third of the adult population, with comparable estimates reported across population-based as well as clinic-based investigations (5). Although MGD is highly prevalent and can be reliably diagnosed based on objective clinical signs regardless of symptom presence, most epidemiological studies do not distinguish between symptomatic and asymptomatic MGD, potentially underestimating the true burden of subclinical disease and limiting insight into the early stages of MGD (7).

Asymptomatic MGD refers to the presence of structural or functional abnormalities of the meibomian glands in individuals who do not report ocular discomfort or dry eye symptoms (8). Reported prevalence estimates for asymptomatic MGD differ substantially among studies, primarily as a result of the lack of uniform diagnostic criteria and variability in assessment methodologies (9). Existing epidemiological data indicate that the prevalence of MGD is affected by demographic characteristics, including age and ethnicity, with subclinical meibomian gland alterations detectable even among younger individuals (10, 11). These findings support the concept that asymptomatic MGD may represent an early stage within the disease continuum rather than a benign condition (12).

When asymptomatic MGD remains unrecognized and untreated, it may gradually progress to terminal duct obstruction accompanied by qualitative alterations in meibum (e.g., altered lipid composition, increased viscosity, and impaired secretion quality) and quantitative reductions in gland function, including decreased meibum secretion and gland impressibility (13–15). With progressive glandular obstruction, diminished lipid secretion together with alterations in protein components of meibomian gland secretions entering the tear film contribute to tear film instability and facilitate the development of evaporative dry eye disease, even when early clinical symptoms are absent (16–18). Persistent tear-film instability associated with untreated MGD can induce chronic ocular surface inflammation and structural damage, which may reduce responsiveness to therapy at later stages (19, 20).

Accordingly, this systematic review and meta-analysis were undertaken to integrate available epidemiological evidence and to determine the prevalence of asymptomatic MGD among adult populations.

## 2 Materials and methods

### 2.1 Study registration

A systematic review of the literature published up to January 13, 2026, was performed in accordance with the Preferred Reporting Items for Systematic Reviews and Meta-Analyses 2020 (PRISMA 2020) guidelines. The review protocol was prospectively registered in the PROSPERO database (registration number: CRD420261283795) after verification that no comparable systematic reviews had been previously registered or published (21).

### 2.2 Search strategy

A comprehensive systematic literature search was conducted to identify studies reporting the prevalence of asymptomatic MGD in the general population. The following electronic databases were systematically searched: PubMed, Web of Science, Scopus, ScienceDirect, and Google Scholar. All searches were performed in the title, abstract, and keyword fields, unless otherwise specified.

Search strategies were limited to studies published in English and conducted in human populations. Eligibility was restricted to original research articles published in peer-reviewed journals. No limitations were applied regarding the year of publication. The literature search was carried out between September 2025 and January 2026, with the final update completed on January 4, 2026.

To establish appropriate search terms, a preliminary exploratory search was conducted to identify relevant keywords appearing in the titles and abstracts of studies focused on asymptomatic MGD. Based on this initial screening, the final search strategy incorporated combinations of the following terms: “meibomian gland dysfunction,” “MGD,” “asymptomatic,” “asymptomatic MGD,” and “asymptomatic meibomian gland dysfunction.” Boolean operators (“AND,” “OR”) were used as appropriate to optimize sensitivity.

### 2.3 Eligibility criteria, study selection, and data collection

Table 1 summarizes the eligibility criteria applied for study selection based on the Population, Exposure, Comparator, Outcome, and Study Design (PECOS) framework. The population included the general adult population, while studies conducted exclusively in children or adolescents were excluded. The exposure of interest was asymptomatic MGD, whereas studies focusing solely on symptomatic MGD or interventional treatments were excluded. No comparator was defined. The primary outcome of interest was the prevalence of asymptomatic MGD, expressed as the proportion of asymptomatic cases within the total assessed population; studies that did not report prevalence data were therefore excluded. Eligible study designs included cross-sectional and observational studies. Reviews, conference abstracts, editorials, commentaries, and publications in languages other than English were excluded from the analysis.

**Table 1:** Evaluation of the certainty of evidence using GRADE framework.

Outcome	Study design	Risk of bias	Inconsistency	Indirectness	Imprecision	Publication bias	Certainty of evidence
Pooled prevalence of asymptomatic MGD	Meta-analysis of observational studies	Low	Serious	Not serious	Serious	Not assessed (*n* < 10)	Low

Study selection and data collection procedures were carried out in compliance with the PRISMA guidelines (22). Two independent researchers performed a standardized literature search across all selected databases. After the literature search was completed, all records were exported to Mendeley Reference Manager, where duplicate records were identified and removed (Zh. A. and I. K.). Study selection and data extraction were performed in accordance with the PRISMA guidelines. Following the removal of duplicates, only unique records were screened for relevance based on their titles and abstracts.

At the final stage of study selection, full-text articles were assessed against the predefined inclusion criteria. Data extraction was performed using a standardized collection form. The extracted variables included the first author, year of publication, country, study design, method used to assess MGD, age group or mean age of participants, diagnostic criteria for asymptomatic MGD, total sample size, and the number of identified asymptomatic MGD cases.

Data extraction was independently conducted by two investigators (Zh. A. and I. K.), after which the extracted datasets were cross-checked and consolidated. Any disagreements related to study selection or data extraction were resolved through discussion with a third reviewer (M. T.), and a high level of agreement (consensus >0.90) was achieved for all included studies.

### 2.4 Meta-analysis

Statistical analyses were conducted using RStudio (version 2025.9.0.387) with R (version 4.5.1; 2025-06-13). Meta-analytical pooling of prevalence estimates was performed using the meta and metafor packages. The overall pooled prevalence of asymptomatic MGD and corresponding 95% confidence intervals (CIs) were estimated using a random-effects model to account for between-study variability. The synthesized results were presented graphically using forest plots. Statistical heterogeneity was assessed using the I^2^ statistic and Cochran’s *Q*-test. To explore potential sources of heterogeneity, additional analyses were conducted, including meta-regression based on the proportion of female participants and the year of publication, as well as influence diagnostics and leave-one-out sensitivity analyses. Publication bias was not assessed because fewer than 10 studies were included in the meta-analysis, rendering such assessments unreliable.

### 2.5 Risk of bias and certainty of evidence

The methodological quality and risk of bias of the included cross-sectional studies were evaluated using a modified version of the Newcastle–Ottawa Scale (NOS) adapted specifically for cross-sectional study designs (23). This modified instrument evaluates study quality across three key domains: Selection (three items), Comparability (one item, with a maximum of two points), and Outcome (two items). Each criterion was scored with up to one point, resulting in a total possible score ranging from 0 to 7. Higher scores indicate greater methodological robustness and a lower risk of bias.

All studies were independently evaluated by two reviewers following prior agreement on the assessment framework. Inter-rater reliability was subsequently determined by a third reviewer. Studies achieving a NOS score of ≥5 were considered to demonstrate acceptable methodological quality and were therefore included in the systematic review.

In accordance with the recommendations of the Cochrane Handbook for Systematic Reviews of Interventions, the certainty of the evidence was evaluated using the Grading of Recommendations Assessment, Development, and Evaluation (GRADE) approach. This evaluation was conducted following established methodological guidance and research notes on the application of GRADE in systematic reviews (24). The certainty of the evidence was quantitatively evaluated using RStudio with the *GRADE* package. This assessment framework encompasses five core domains: risk of bias, evaluated using the previously described Newcastle–Ottawa Scale for cross-sectional studies; inconsistency, quantified using the I^2^ statistic; indirectness, examined according to PICO criteria; imprecision, determined by whether the 95% confidence interval of the pooled estimate encompassed the prespecified threshold of interest; and publication bias, evaluated based on the results of Egger’s regression test.

## 3 Results

### 3.1 Study selection and characteristics of the included studies

A total of 296 records were identified through database searching, including PubMed (*n* = 44), Web of Science (*n* = 53), Scopus (*n* = 47), ScienceDirect (*n* = 23), and Google Scholar (*n* = 129). Following the elimination of 128 duplicate entries, 168 unique records were retained for title and abstract screening, during which 140 records were excluded.

In total, 28 articles were identified for full-text evaluation. Full-text versions were unavailable for two reports; consequently, 26 articles underwent eligibility assessment. After detailed full-text review, 18 reports were excluded for the following reasons: review articles (*n* = 9), absence of prevalence data on asymptomatic MGD (*n* = 4), inclusion of symptomatic MGD only (*n* = 2) (25, 26), studies conducted in children populations (*n* = 1) (27), and meeting abstracts (*n* = 2).

**Figure 1:**
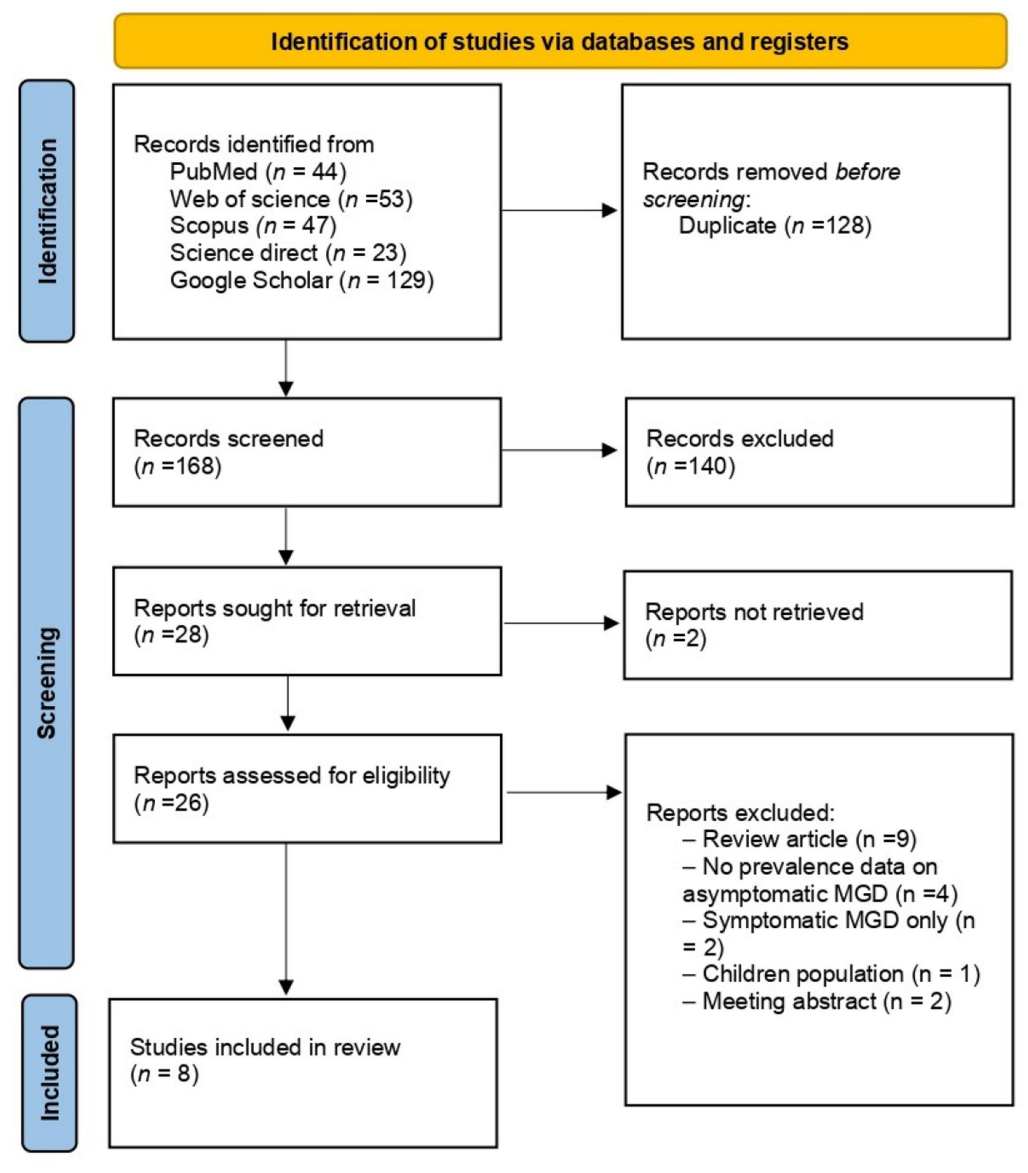
PRISMA flowchart of study inclusion.

Ultimately, 8 studies satisfied the inclusion criteria and were incorporated into the final review. The process of study identification and selection is depicted in the PRISMA flow diagram (Figure 1) (22).

The included studies dated from 2012 to 2023, were exclusively cross-sectional in design, including both population-based and clinic-based cross-sectional investigations. The studies were conducted across multiple geographic regions, encompassing East Asia (Japan, Taiwan), Europe (Spain), Africa (Ghana), North America (Canada), and Oceania (Australia), with East Asia and Europe being the most frequently represented regions.

The mean age of participants varied widely, ranging from young adults (21.9 ± 3.8 years) to elderly populations (71.1 ± 8.5 years). Sample sizes ranged from 40 to 1,329 participants. Across all studies, a total of 3,637 participants were assessed, with 1,313 cases of asymptomatic MGD identified.

Female participants predominated in most studies. In all included studies, symptom evaluation and the classification of asymptomatic MGD were uniformly based on the Ocular Surface Disease Index (OSDI) questionnaire. A comprehensive summary of the characteristics of the included studies is provided in Table 2.

**Table 2:** Description of the included studies.

Last name, year	Study design	Country	Age	Population size	Asymptomatic MGD cases	Number of female participants	Questionnaire used
Amano and Inoue (40)	Cross-sectional study	Japan	71.1 ± 8.5	510	242	305	OSDI
Asiedu et al. (41)	Cross-sectional study	Ghana	21.9 ± 3.8	215	21	8	OSDI
Chen et al. (42)	Cross-sectional study	Taiwan	49.0 ± 10.4	1,329	89	12	OSDI
Chiou et al. (43)	Cross-sectional study	Taiwan	64.0 ± 12.6	153	87	52	OSDI
González-Cavada et al. (44)	Cross-sectional study	Spain	27.5	50	50	24	OSDI
Ngo et al. (45)	Cross-sectional study	Canada	62 ± 15	40	20	20	OSDI
Viso et al. (46)	Cross-sectional study	Spain	63.4 ± 14.5	1,155	619	390	OSDI
Yeotikar et al. (28)	Cross-sectional study	Australia	43.9 ± 11.8	185	185	109	OSDI

MGD, meibomian gland dysfuncton; OSDI, ocular surface disease index.

### 3.2 Meta-analysis results

**Figure 2:**
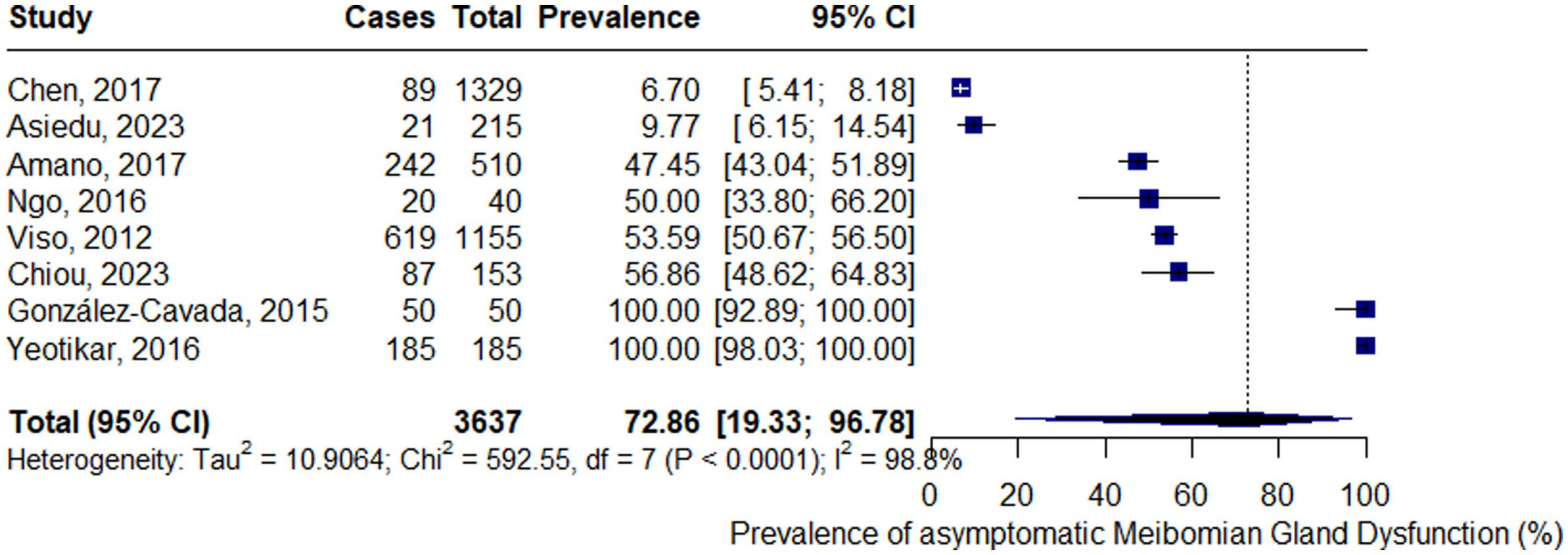
Forest plot of the pooled prevalence of asymptomatic meibomian gland dysfunction.

The overall prevalence of asymptomatic MGD was 72.86% (95% CI: 19.33–96.78%), as estimated using a random-effects meta-analysis of proportions with logit transformation. The corresponding prediction interval was wide (6.7–100%). Consequently, a considerable between-study heterogeneity was observed, with an I^2^ value of 98.8%, and a between-study variance (τ^2^) of 10.91. Heterogeneity was further confirmed by Cochran’s *Q*-test (*Q* = 592.55, df = 7, *p* < 0.0001). The forest plot of the pooled prevalence of asymptomatic MGD is presented in Figure 2.

**Figure 3:**
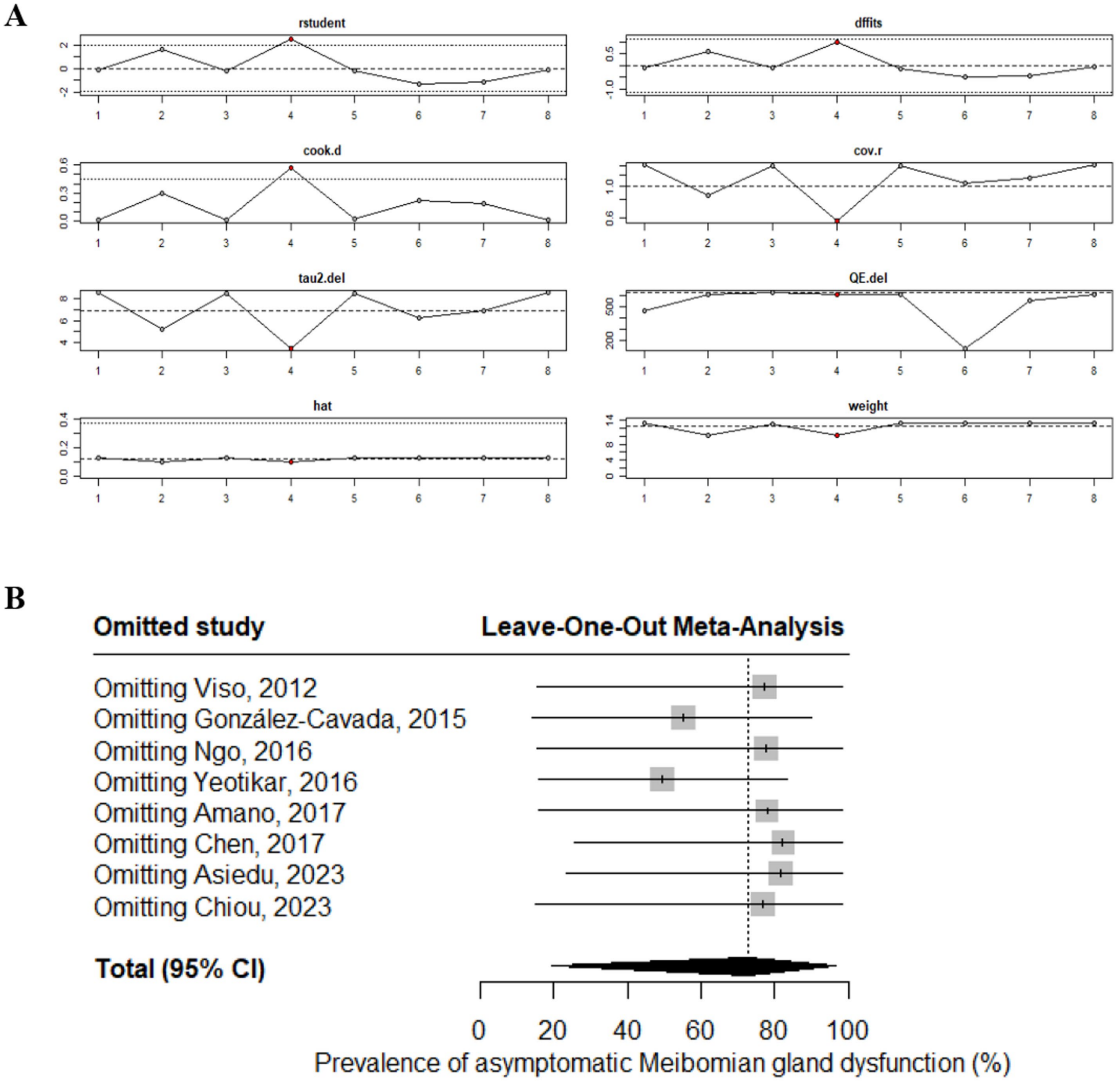
Heterogeneity assessment of the pooled prevalence of asymptomatic meibomian gland dysfunction. **(A)** Influence analysis. **(B)** Leave-one-out analysis.

Heterogeneity in the pooled prevalence estimate was assessed using influence diagnostics and leave-one-out sensitivity analyses. The influence analysis identified one study.

with relatively greater impact—Yeotikar (28)—as indicated by Cook’s distance. Consistent with this finding, the leave-one-out analysis revealed that sequential exclusion of Yeotikar (28) study resulted in the most substantial change to both the pooled estimate and its confidence interval, as presented in Figures 3A,B.

**Figure 4:**
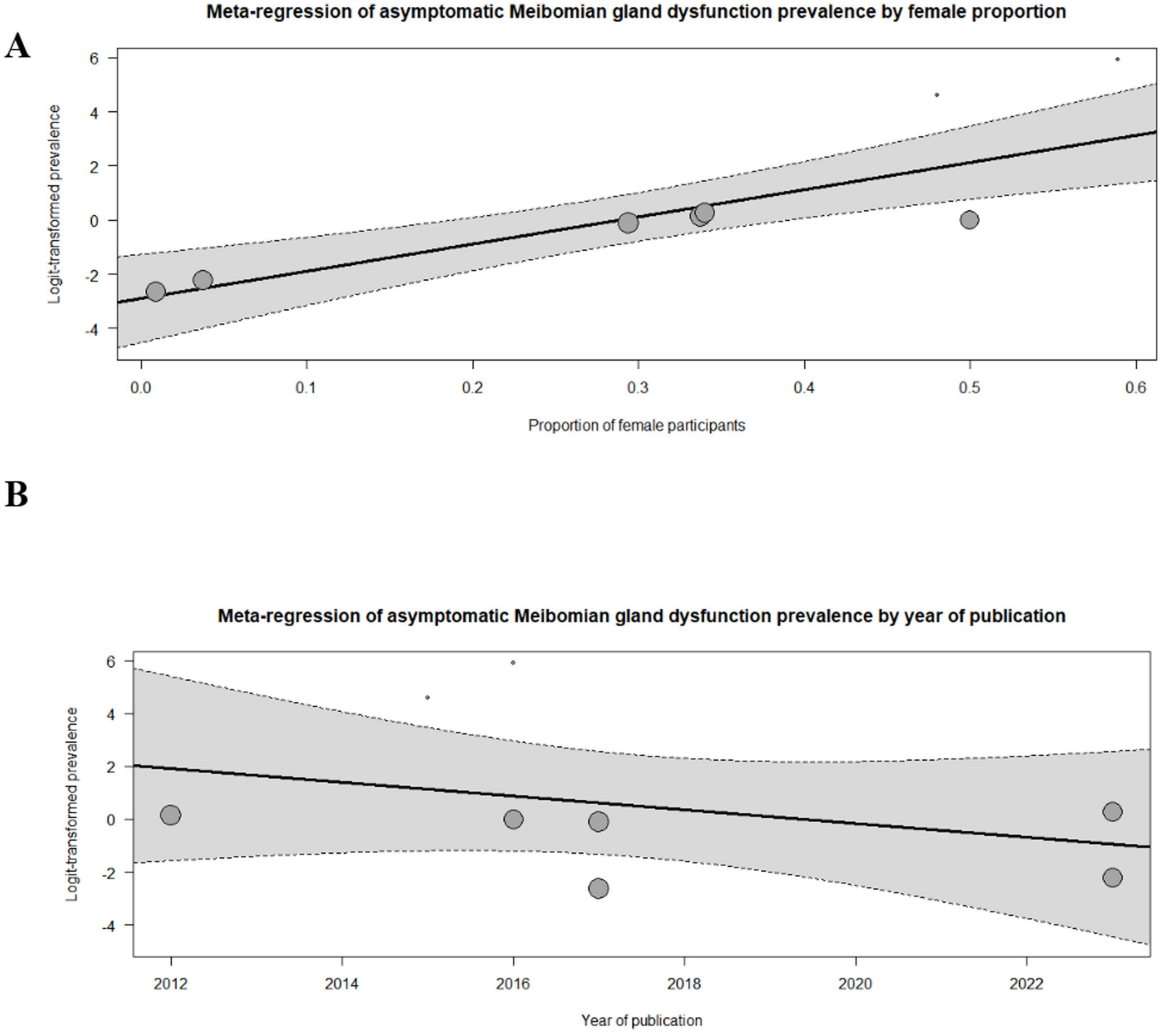
Meta-regression of asymptomatic Meibomian gland dysfunction by. **(A)** Proportion of female participants. **(B)** Year of publication.

Furthermore, two meta-regression models were conducted to explore potential sources of heterogeneity in the pooled prevalence of asymptomatic MGD. The first model assessed the association between the proportion of female participants and prevalence estimates, while the second model examined the effect of year of publication. The meta-regression analyses indicated that sex distribution contributed to between-study variability (*p* < 0.001), whereas no temporal trend in prevalence estimates was observed (*p* = 0.33), as presented in Figures 4A,B.

Substantial heterogeneity was observed across the included studies (*I*^2^ = 98.8%). To explore potential sources of heterogeneity, meta-regression analyses were performed using the proportion of female participants and year of publication as covariates. However, neither variable showed a statistically significant association with the pooled prevalence estimate. Influence analyses and leave-one-out sensitivity analyses demonstrated that no single study disproportionately affected the overall results, indicating that the pooled estimate was stable despite the high heterogeneity.

The GRADE assessment of certainty of evidence (Table 1) indicated that the certainty for the pooled prevalence of asymptomatic MGD was low. This assessment was driven primarily by the observational design of the included studies and the presence of substantial unexplained between-study heterogeneity. Accordingly, the pooled prevalence estimate should be interpreted with appropriate caution.

## 4 Discussion

This systematic review and meta-analysis demonstrates that asymptomatic MGD is common among adults, with a pooled prevalence estimate of 72.86% (95% CI: 19.33–96.78%). This finding highlights the substantial burden of subclinical MGD and suggests that a large proportion of adults may harbor meibomian gland abnormalities in the absence of overt ocular symptoms.

Meibomian glands play a central role in maintaining tear film stability by secreting lipids that reduce tear evaporation (29, 30). The high burden of asymptomatic MGD has important implications for the pathogenesis and prevention of dry eye disease. Subclinical gland dysfunction, even when not accompanied by symptoms, may compromise the lipid layer of the tear film, increase evaporative stress, and promote low-grade inflammation of the ocular surface. Data from a population-based cohort in northern China demonstrated a higher prevalence of asymptomatic compared with symptomatic MGD, at 21.9 and 8.6%, respectively (31). Over time, these alterations may predispose individuals to the development of symptomatic dry eye syndrome, particularly in the presence of additional risk factors such as aging, hormonal changes, prolonged screen use, or environmental stressors (32, 33).

Given the high prevalence of asymptomatic MGD, early identification should be prioritized in clinical practice. Objective diagnostic tools, including lipid interferometry, slit-lamp assessment of eyelid anatomy, evaluation of gland expressibility and secretion quality, and meibography, may enable detection of subclinical disease independent of symptoms. Incorporating these assessments into routine eye examinations could facilitate timely preventive interventions before irreversible gland damage occurs (34). Such an approach reflects a transition from reactive treatment of established dry eye disease to proactive prevention and was examined in a study population with a mean age of 46.2 ± 14.8 years, in which women constituted 77.4% and participants of Chinese origin 87.1%. The average OSDI score was 35.2 ± 21.7 (29).

Meta-analytic estimates indicate that the overall pooled prevalence of MGD is approximately 35.8%, with consistently similar prevalence rates reported in both clinical settings (35.8%) and population-based studies (35.9%). A modest but statistically significant sex-related difference has been observed, with a higher likelihood of MGD among men compared with women (OR 1.24, 95% CI 1.01–1.52; *p* = 0.034). Substantial geographic variation has also been documented, with prevalence ranging from approximately 21–30% in African and Caucasian populations to markedly higher rates in Arab (71.0%) and Hispanic (67.5%) populations (5).

These findings support strategies aimed at improving meibomian gland secretion quality and stabilizing tear film homeostasis to delay or prevent progression to symptomatic disease; however, the referenced study was limited to symptomatic patients, among whom signs of MGD were observed in 70.3% of the population with a mean age of 55.4 ± 16.6 years, whereas our analysis indicates a substantially higher prevalence of asymptomatic MGD, underscoring the importance of early identification prior to symptom onset (25). Consequently, addressing gland dysfunction at an early stage may reduce chronic inflammation, preserve glandular architecture, and maintain ocular surface integrity—an approach that is particularly relevant given the therapeutic challenges and persistent symptom burden associated with advanced MGD and dry eye disease (35).

From a broader clinical and public health perspective, the high prevalence of asymptomatic MGD suggests that a large proportion of the adult population may be at risk for future dry eye disease without being aware of it. Early diagnosis and preventive management could reduce the long-term burden of dry eye syndrome, including healthcare utilization, treatment costs, and productivity loss (36–38). These considerations are especially important in aging societies, where both MGD and dry eye disease are expected to increase in prevalence. By 2030, one in six people in the world will be aged 60 years or older, and by 2050 the proportion of the global population over 60 will nearly double from 12 to 22%. In addition, by 2050 one in six people globally will be over age 65, reflecting a dramatic rise in the elderly population (39).

In summary, the findings of this meta-analysis highlight that asymptomatic MGD is not a rare or incidental condition but a common and clinically relevant entity. Recognizing asymptomatic MGD as an early stage in the continuum of ocular surface disease supports the rationale for early diagnosis and timely intervention. Proactive management of asymptomatic MGD may represent an effective strategy to prevent or delay the onset of dry eye syndrome and to preserve long-term ocular surface health.

### 4.1 Limitations

All studies included in the analysis employed observational cross-sectional designs, which may be associated with selection bias and consequently constrain the overall strength of the evidence. In addition, only eight studies met the inclusion criteria, which may limit the robustness and generalizability of the pooled prevalence estimates. Because fewer than 10 studies were included, formal assessment of publication bias (e.g., funnel plot or Egger’s regression test) was not performed in accordance with methodological recommendations; however, the possibility of publication bias cannot be entirely excluded.

Although the present analysis concentrated on pooled prevalence estimates, it was not possible to adjust for clinical heterogeneity—such as variations in diagnostic definitions, methods of meibomian gland evaluation, or disease severity—because of inconsistent reporting across the included studies. As a result, the potential influence of underlying anatomical changes or early inflammatory alterations on asymptomatic MGD prevalence could not be reliably evaluated. Substantial heterogeneity (*I*^2^ = 98.8%) remained across studies, and although meta-regression partially explained variability related to sex distribution, residual heterogeneity is likely attributable to unmeasured factors such as age distribution, environmental exposure, and examiner-dependent assessment techniques.

Given these limitations, the observational nature of the evidence, and the low certainty of evidence, the pooled prevalence estimates should be interpreted with caution, underscoring the need for standardized diagnostic approaches and well-designed prospective studies to better define the role of early detection and preventive treatment of asymptomatic MGD in reducing the risk of dry eye disease.

### 4.2 Future research directions

- Early preventive interventions targeting individuals with asymptomatic MGD should be evaluated to determine their effectiveness in delaying or preventing the onset of clinically manifest dry eye disease.
- Future research should incorporate stratified analyses by sex, age, and geographic region, considering the identified contribution of sex distribution to variability in prevalence estimates.

## 5 Conclusion

This systematic review and meta-analysis demonstrate that asymptomatic MGD is highly prevalent in adult populations, with a pooled prevalence of 72.86% across eight cross-sectional studies including 3,637 participants. Our findings indicate that meibomian gland abnormalities are common even in the absence of ocular symptoms. These results are clinically relevant, as asymptomatic MGD may represent an early stage in the disease continuum that precedes the development of dry eye syndrome. Early identification of MGD may enable timely preventive management, potentially reducing disease progression and the need for prolonged or intensive treatment of dry eye disease. From a health system perspective, earlier diagnosis and intervention may also help decrease long-term healthcare costs associated with chronic management of dry eye syndrome.

## Data Availability

The original contributions presented in the study are included in the article/Supplementary material, further inquiries can be directed to the corresponding author.

## Supplementary material

Supplementary Table S1 Detailing the search strategy applied in the systematic review.

Supplementary Table S2 Outlining the study inclusion and exclusion criteria based on the PICOS framework.

Supplementary Table S3 Presenting the risk of bias (quality) assessment conducted using the Newcastle-Ottawa Scale.
