# Supplementary material for: Prevalence of asymptomatic meibomian gland dysfunction in the general adult population: a systematic review and meta-analysis
Source: Front Med (Lausanne). 2026 Apr 20;13:1797225. doi: 10.3389/fmed.2026.1797225 (PMC13137810; doi:10.3389/fmed.2026.1797225)
Supplement: Supplementary file 1 [file Data_Sheet_1.zip › S1 Table. Search strategy.docx]

**Table S1. Search strategy of the Systematic Review of the Prevalence of asymptomatic MGD in general population**

| **Pubmed** | **Web of Science** | **Scopus** | **Science Direct/or other** | **Google Scholar** |
| --- | --- | --- | --- | --- |
| **Criteria: Search in All Fields.**  **Filter: Language: English, Species: Human**  **No filter on the year of publication**  **Search date: 04.01.2026**   1. Meibomian Gland Dysfunction asymptomatic - 44 | **Criteria: Search in All Fields.**  **Filter: include only “Articles”**  **Search date: 04.01.2026**   1. meibomian gland dysfunction AND asymptomatic  - 53 | **Criteria: Search in All Fields.**  **Filter: Language: English,**  **Filter: include only “Articles”**  **Search date: 04.01.2026**   1. Meibomian Gland Dysfunction asymptomatic - 47 | **Criteria: Search in All Fields.**  **Filter: Language: English,**  **Filter: include only “Academic Journals”**  **Search date: 04.01.2026**   1. ''asymptomatic Meibomian Gland Dysfunction '' "asymptomatic MGD"  - 23 | **Criteria: Search in Title only Fields.**  **Search date: 04.01.2026**   1. asymptomatic MGD"  - 109  1. "asymptomatic meibomian gland dysfunction" - 20 |
